# Military Hospital Construction

**Published:** 1916-11-04

**Authors:** W. J. Simpson

**Affiliations:** Professor of Hygiene and Public Health in the University of London, King's College.


### THE EDITOR'S LETTER-BOX.

### Military Hospital Construction.

*To the Editor of* The Hospital.

Sir,—I have much pleasure in giving you my opinion on the closed wards erected by the Barrack Department in Leicester and on the open wards built by the Leicester authorities. After personal inspection, I have no hesitation in stating that the open wards are airy, cheerful, comfortable, and healthy, and that the construction, though it may be improved so as to afford better shelter in exceptionally stormy weather, is on sound hygienic principles. The same cannot be said of the closed wards erected by the Barracks Department. On the contrary, they are of a retrograde type and in every way unsuitable for the treatment of soldiers. The closed wards of this type are a return to the pre-Crimean arrangements, and not in consonance with the principles relating to modern hospitals. Light, ventilation, and floor space are inadequate, and the arrangements generally are such that it may be safely stated that no medical officer specially acquainted with the requirements of modern hospital construction or any well-known and experienced hospital architect was consulted. The wards are 150 feet long and 40 feet wide, containing four rows of beds—100 in all—and are constructed on a plan condemned by Miss Florence Nightingale in 1863 in her " Notes on Hospitals." I consider it unfair to the sick and wounded soldiers, who deserve the very best accommodation, to be placed under conditions which must materially retard recovery and interfere with the full benefits to be derived from skilled medical and surgical treatment.

I hope your article drawing attention to the subject will be the means of ending this retrogressive movement and reinstate the open wards, which are undoubtedly superior to the closed wards as military emergency hospitals.—Yours truly,

(Signed) W. J. Simpson, M.D., F.R.C.P.,
Professor of Hygiene and Public Health in the University of London, King's College.

